# Materializing the international migrant health policy in health sciences curricula in Chile

**DOI:** 10.15649/cuidarte.4435

**Published:** 2025-05-01

**Authors:** Debbie Álvarez-Cruces, Alejandra Nocetti-de-la-Barra, Juan Mansilla-Sepúlveda

**Affiliations:** 1 Universidad de Concepción, Concepción, Chile. E-mail: debbiejalvarez@udec.cl Universidad de Concepción Concepción Chile debbiejalvarez@udec.cl; 2 Universidad Católica de la Santísima Concepción, Concepción, Chile. E-mail: anocetti@ucsc.cl Universidad Católica de la Santísima Concepción Concepción Chile anocetti@ucsc.cl; 3 Universidad Católica de Temuco, Temuco, Chile. E-mail: jmansilla@uct.cl Universidad Católica de Temuco Temuco Chile jmansilla@uct.cl

**Keywords:** Health Sciences, Professional Training, Migrants, Policy, Cultural Competence, Intercultural Competence, Ciencias de la Salud, Formación Profesional, Migrantes, Políticas, Competencia Cultural, Competencia Intercultural, Ciências da Saúde, Capacitação Profissional, Políticas, Competência Cultural, Competência Intercultural

## Abstract

**Introduction:**

In Chile, the International Migrant Health Policy (PSMI in Spanish) mandates the inclusion of topics such as migration, interculturality, human rights, social determinants of health, and gender in higher education curricula. However, it is unknown whether this effectively happens.

**Objective:**

To understand the materialization of the PSMI in health sciences curricula.

**Materials and Methods:**

This interpretative/hermeneutic study included semi-structured interviews with program directors, academic faculty, clinical professors, and students. It also involved a document analysis across different health sciences degree programs at three regional universities in Chile. Data analysis included open, axial, and selective coding with ATLAS.ti version 24.

**Results:**

A total of 179 informants participated. Three main categories emerged: Health Institution Setting, University Institution Setting, and Degree Program Setting, each comprising facilitating or hindering subcategories for materializing policy materialization. The hermeneutic analysis made it possible to interrelate these subcategories, producing a semantic network to understand the phenomenon. Facilitators were scarce and isolated from the network core, while hindrances were more numerous, cohesive, and robust, reinforcing an ethnocentric model of professional education validated by ethnocentric healthcare practices in clinical settings.

**Discussion:**

The concept of “cultural blinders” is proposed in place of “cultural blindness” as there is awareness of the cultural influences on healthcare that are nonetheless not integrated.

**Conclusion:**

Current curricula do not align with the PSMI. Coordinated policies between the Ministry of Education and the Ministry of Health are imperative to reverse the existing healthcare status quo.

## Resumen

**Introducción:**

En Chile, la Política de Salud de Migrante Internacional establece la incorporación curricular de temáticas sobre migración, interculturalidad, derechos humanos, determinantes sociales y género en la Educación Superior; pero, se desconoce si esto ocurre de manera efectiva.

**Objetivo:**

Comprender la concreción de la Política de Salud de Migrante Internacional en el currículum de Ciencias de la Salud.

**Materiales y métodos:**

Estudio Interpretativo/Hermenéutico que incluyó entrevistas semiestructuradas a jefes de carrera, académicos- docentes, guías clínicos y estudiantes, además de análisis documental de diferentes carreras de la salud de tres Universidades regionales de Chile. El análisis de datos incluyó codificación abierta, axial y selectiva con la asistencia del programa ATLAS.ti 24.

**Resultados:**

Participaron 179 informantes. Se conformaron tres categorías: Ámbito Institución de Salud, Ámbito Institución Universitaria y Ámbito de la Carrera, cada una con subcategorías facilitadoras u obstaculizadoras para la concreción de la política. El análisis Hermenéutico permitió interrelacionar las subcategorías para comprender el fenómeno global con una red semántica. Los facilitadores fueron escasos y aislados de la red. Los obstaculizadores están en mayor cantidad, más cohesionados y robustos, originando una formación profesional etnocéntrica que se valida con una atención de salud etnocéntrica en los campos clínicos.

**Discusión:**

Se propone el concepto de Gríngola Cultural en vez de Ceguera Cultural, porque existe consciencia de los factores culturales que influyen en la atención de salud, pero no se incorporan.

**Conclusiones:**

El currículum no responde a la Política de Salud de Migrante Internacional, es imperativo contar con políticas mancomunadas entre el Ministerio de Educación y Ministerio de Salud que reviertan el statu quo existente en la estructura sanitaria.

## Resumo

**Introdução:**

No Chile, a Política de Saúde do Migrante Internacional (PSMI) estabelece a incorporação curricular de temáticas como migração, interculturalidade, direitos humanos, determinantes sociais e gênero na educação superior; porém, não se sabe se isso ocorre de forma eficaz.

**Objetivo:**

Compreender a implementação da PSMI no currículo de Ciências da Saúde.

**Materiais e métodos:**

Estudo interpretativo/ hermenêutico que incluiu entrevistas semiestruturadas com diretores de programas, professores acadêmicos, guias clínicos e alunos, além de análise documental de diferentes programas de saúde de três universidades regionais do Chile. A análise de dados incluiu codificação aberta, axial e seletiva com o auxílio do programa ATLAS.ti 24.

**Resultados:**

Participaram 179 informantes. Foram estabelecidas três categorias: Escopo da Instituição de Saúde, Escopo da Instituição Universitária e Escopo da Carreira, cada uma com subcategorias que facilitam ou dificultam a implementação da política. A análise hermenêutica permitiu que as subcategorias fossem inter-relacionadas para compreender o fenômeno global com uma rede semântica. Os facilitadores eram poucos e isolados da rede. Os obstáculos são mais numerosos, mais coesos e mais robustos, dando origem a uma formação profissional etnocêntrica que é validada por cuidados de saúde etnocêntricos em ambientes clínicos.

**Discussão:**

O conceito de Gríngola Cultural é proposto em vez de Cegueira Cultural, porque há consciência dos fatores culturais que influenciam os cuidados de saúde, mas eles não são incorporados.

**Conclusões:**

O currículo não responde ao PSMI; é imperativo que haja políticas conjuntas entre o Ministério da Educação e o Ministério da Saúde que revertam o status quo existente na estrutura da saúde.

## Introduction

During the 20th century, following the two World Wars, migration was largely characterized by the movement of people from one nation-state to another, predominantly from the Global South to the Global North and from East to West^1^. Countries known as developed countries become destinations for foreigners from nations categorized as underdeveloped. However, since the 1970s, there has been a notable rise in South-South migration trends^2^. 

In Chile, this reality became evident in 1990, coinciding with the country's return to democracy. At that time, Chile gained international recognition for achieving a degree of political, economic, and administrative stability, which sparked interest and expectations among South American populations seeking to reside in the country^2^,^3^. Since then, the proportion of Latin American immigrants has grown from 0.7% to 7.7% by the end of 2022, placing Chile above the global average and ranking it second in Latin America after Costa Rica^4^. 

It is well known that the migrant population experiences heightened vulnerability throughout the migration cycle, which includes the decision to migrate, the journey itself, settlement in the destination country, and potential return^1^,^5^,^6^. Recognizing these difficulties and the right to health as a fundamental human right, Chile established a series of regulations and decrees aimed at improving healthcare access for this group of people, beginning in 2003 with the protection of pregnant women and children^7^,^8^. However, in other areas such as employment, wages, housing, education, and social rights, there has been limited organization, coordination, and continuity in policies, placing migrants' living conditions at risk and affecting their health^8^,^9^. 

In addition to the above, cultural factors, such as beliefs, habits, customs, and traditions that influence individuals' decision-making regarding health and disease processes, should also be considered^10^,^11^. For this reason, interprofessional collaboration is required to address the multicausal nature of the factors present, along with healthcare personnel who are properly trained to understand, empathize with, and provide culturally appropriate care. 

This is why international organizations stress the importance of developing intercultural competence in healthcare personnel. They suggest forming partnerships with health sciences education institutions to support the materialization of current policies related to human rights (HR), social justice, equity, and inclusion in healthcare^12^-^14^.

In the health field, the concepts "cultural competence," "intercultural competence," and "transcultural competence" are often used interchangeably^15^. However, current recommendations from philosophical, anthropological, and sociological disciplines indicate that "intercultural competence" (ICC) is the most appropriate construct. It refers to developing knowledge, attitudes, and skills to address the cultural needs of others^15^,^16^. 

Today, many models and definitions of ICC exist within the health field. However, they all recognize the importance of developing cultural self-awareness as a foundation for accepting, understanding, and respecting cultural diversity^17^. In addition, they highlight the need to cultivate skills for delivering culturally relevant care—care that acknowledges and integrates an individual’s cultural background, live perspective, health-illness concepts, and healing systems^18^. 

In Chile, although the arrival of foreign nationals began in the late 20th century, the International Migrant Health Policy (PSMI in Spanish) was not established until 2017. Its guidelines and strategies promote the inclusion of topics related to migration and health, interculturality, human rights, social determinants of health, and gender in the curricula of universities and technical training institutes^7^. 

Countries that have historically been destinations for migrants, such as Canada, England, the United States, and Australia, have implemented mandatory accreditation policies and criteria for health-related programs to develop ICC in professional training^19^. These contents are integrated either progressively or as elective components across different subjects within different degree programs^20^,^21^. 

Theoretical and empirical evidence shows that developing ICC in healthcare professionals leads to better health outcomes, increased patient satisfaction, and reduced disparities in care. On the contrary, the absence of ICC training contributes to multiple issues, including lack of cultural sensitivity, culturally and linguistically inappropriate services, lower quality of care, poor adherence to treatment, and difficulties in the professional-patient relationship^17^,^22^,^23^. 

Several studies conducted in different countries have assessed the extent to which intercultural competence (ICC) is integrated into health curricula^24^. However, in Chile, the incorporation of ICC remains scarce^25^,^26^. A similar reality was observed in countries with a longer history of ICC in healthcare, where its inclusion in professional training was implemented late^27^. In addition, it is necessary to consider that national and international research shows different forms of discrimination against migrants in healthcare settings^26^,^28^-^30^. Such experiences can provoke feelings of distress, rejection, frustration, helplessness, and disappointment among health sciences students^26^,^31^,^32^. However, these behaviors and malpractices may also result in vicarious learning perpetuated by future generations^28^. 

Given the above, it is relevant to understand the materialization of the PSMI in health sciences curricula. 

## Materials and Methods

It is a qualitative study with an interpretative/hermeneutic approach as it delves into the conditions, context, and meanings that enable an understanding of the phenomenon through the fusion of horizons based on individuals' intersubjective and symbolic interactions^33^. From this perspective, an objective description of facts alone is not enough. The researcher is called to interpret and construct reality based on the analysis of the information provided by the participants, according to their worldview^34^. In this way, the understanding of meaning allows the global phenomenon to be understood through a coherent interpretation^33^. 

A multiple case study design was employed, characterized by exploring a broad phenomenon with several interconnected cases. This approach requires diverse contexts and multiple sources of information to achieve an understanding of the phenomenon^35^. Three higher education institutions were included, located in different regions of Chile and distant from each other. These institutions offer different health sciences programs, including nursing, chemistry and pharmacy, kinesiology, medicine, nutrition, midwifery, and dentistry. 

Participants included individuals involved in the teaching and learning process within health sciences professional training programs. They were selected using different sampling strategies based on Patton's classification^34^. Program directors (PD) were considered key informants; academic faculty (AF) and clinical professors (CP) were selected as typical cases; and students were chosen through intensity sampling. Semi-structured interviews were conducted to explore the materialization of the PSMI in the curricula. As the interviews progressed, Institutional Graduate Profile (IGP), Program-specific Graduate Profile (PGP), and course syllabi were requested to support document analysis and verify the presence or articulation of content related to the PSMI within the health sciences curricula. 

Participants were contacted by email, which included a detailed explanation of the research objective and scope, along with the informed consent form. Once the signed consent was received, interviews were conducted through the Zoom platform. Audio recordings of the interviews were saved for subsequent transcription. 

To meet the quality^36^, credibility, and confirmability criteria, fieldwork was conducted over an extended period of time until data saturation was achieved for each degree program and university. Initial findings were validated with the participants. Regular meetings were held with the research team, and data triangulation was completed by source (participant and documents). 

Transferability was supported through extensive data collection at geographically distant universities. Additionally, different sampling strategies were used depending on the participant group, with inclusion and exclusion criteria outlined in Table 1. Dependability was ensured through independent data analysis conducted by each researcher, followed by comparison, discussion, and unification of opinions. 

**Table 1:** Inclusion and exclusion criteria for participants

Participant	Inclusion criterion	Exclusion criterion
Program director	Professionals working in the fields of medicine, nursing, kinesiology, midwifery, chemistry and pharmacy, nutrition, or dentistry at public or private universities.	Professionals who are foreign nationals or who self-identify with any ethnic group. In such cases, the Dean or Vice- Dean will be interviewed instead.
Health sciences students	Students enrolled in medicine, nursing, kinesiology, midwifery, chemistry and pharmacy, nutrition, or dentistry at public or private universities who are in their final year of training or completing supervised rotations.	Students who are foreign nationals or who self-identify with any ethnic group. Students with previous degrees in social sciences. Students with prior degrees in health sciences who have already practiced professionally.
Academic faculty	Professionals teaching in medicine, nursing, kinesiology, midwifery, chemistry and pharmacy, nutrition or dentistry programs at public or private universities, and who address migration-related topics in their teaching.	Professionals who are foreign nationals or who self-identify with any ethnic group.
Clinical professors	Professionals in medicine, nursing, kinesiology, midwifery, chemistry and pharmacy, nutrition, or dentistry with at least 2 years of experience in caring for migrant patients and who currently supervise or have supervised students on rotations.	Professionals who are foreign nationals or who self-identify with any ethnic group.

The research was approved by two accredited Chilean scientific committees. The protocol established strict confidentiality for participants, the data, and the collaborating universities and healthcare institutions to prevent stigmatization. 

Data analysis was carried out using ATLAS.ti version 24. The process followed the stages of open, axial, and selective coding as outlined in Grounded Theory^37^. Open coding and axial coding were conducted inductively, and several in vivo codes derived from the information provided by the participants were used to develop categories and subcategories. Selective coding enabled the identification of a central concept or theme, which informed the creation of a comprehensive diagram illustrating the relationships among the subcategories. It is necessary to mention that this study is part of a broader research conducted for a doctoral dissertation. The dataset related to this study is available in the Figshare repository^38^. 

## Results

A total of 179 informants participated: 106 students (S), 15 program directors (PD), 28 academic faculty (AF), and 30 clinical professors (CP) Table 2.

**Table 2:** Participant characteristics by type, sex, and university institution

	Universities						
Degree programs by institution	Institution 1		Institution 2		Institution 3		
	4		7		4		Total by type
Participants / Sex	W	M	W	M	W	M	
Students	21	10	38	23	11	3	106
Program directors	3	1	7	0	3	1	15
Academic faculty	7	0	10	4	6	1	28
Clinical professor	4	1	13	4	7	1	30
Total by sex	35	12	68	31	27	6	
Total by institution	47		99		33		
Total participants	179						

W: Woman, M: Men

The qualitative analysis began with a descriptive analysis that led to the identification of three main categories corresponding to the different settings in which students engage throughout their professional training. One of them was the Health Institution Setting (HIS), referring to the clinical environments where students conduct their clinical rotations (Table 3). Another setting was the University Institution Setting (UIS), which encompasses the general guidelines that shape each program's curriculum (Table 4). The last setting was the Degree Program Setting (DPS), where the teaching-learning process occurs within specific courses (Table 5). Within each category, subcategories were then identified and classified as either facilitators or hindrances to materializing the PSMI. 

The nomenclature used for textual citations followed this structure: the first letter indicates the participant's sex (W: woman, M: man); the second element refers to the type of participant (S: student, PD: program director, AF: academic faculty, and CP: clinical professor); the third component represents the academic program (N: nursing, QF: chemistry and pharmacy, K: kinesiology, M: medicine, Nu: nutrition, Mi: midwifery, and D: dentistry); the first number refers to the participant's number within their degree program; and the number in parentheses identifies the university. 

**Table 3:** Categories and subcategories related to the materialization of the International Migrant Health Policy in the Health Institution Setting

Category 1: Health Institution Setting			
Subcategories	Citation Operational	Operational description	Verbatim quotation
Facilitators for materialization			
1. Development of knowledge and practical skills	184	Knowledge and skills acquired by the students while caring for migrant patients in clinical settings	When it was our turn for clinical rotations, that was when the students had to care for migrant women. Usually, what I do is assign each student a group of patients, and they work to meet their needs in a comprehensive way. You even felt proud of the effort they put in because the truth is that they even coordinated with the social worker to connect patients to support networks and educate them about these networks. WAFMi1(3)
2. Positive role modeling at work	50	Learning through positive role modeling observed by students while caring for migrant patients in clinical settings	The midwives played an important role during that final-year clinical rotation. They seemed to know a bit more about this [caring for migrant patients], and they guided us a little more. It was like they already knew what the patients were going through—even if the patients didn’t speak Spanish, they knew how to interpret what they were feeling. That was good. They’d clearly seen many more patients than we had. In the end, they were more of a guide to us than the doctors. MSM2(1)
Hindrances for			
1. Discrimination against migrants during healthcare	301	Different forms of discrimination observed by students while caring for migrant patients in clinical settings	What happens is that just being a foreigner —whether Venezuelan, Bolivian or from any other country—no matter your social status, just because you are a foreigner, you’re discriminated against in Chilean healthcare. Patients could have all kinds of academic degrees, amazing jobs, but they’re still looked down on. There’s this sort of collective rejection. In the hospital, you can hear it in the comments people make. It’s not outright discrimination—it’s more subtle, underhanded. WSMi6(2)
2. Ethnocentric healthcare	263	Egalitarian care that reflects the dominant care practices of the host country, delivered to migrants without consideration for their cultural backgrounds, as observed by students in clinical settings	Well, when it comes to migrant patients, the fundamental idea is that they should be treated like any other patient; it shouldn't make so much of a difference whether they're Venezuelan or Haitian. The only big difference we've faced is the language, especially with Haitians. But overall, final-year students completing their rotations are told to treat everyone equally, without distinctions (...). I do remind students to be empathetic, but we haven’t had any cases where they’ve lacked empathy. MCPK2(2)
3. Structural aspects in healthcare	168	Protocols, regulations, and standardized procedures established by the Ministry of Health that hinder the provision of culturally sensitive care to migrants, as observed by students in clinical settings	So, I think the barriers are the regulations. The standards say one thing, and the nurses—well, the standard says this and that's done and nothing else. So, I believe students themselves have to start recognizing and normalizing different practices [eating habits of other cultures]. Start reading, start listening. It’s not that what they [migrant patients] do is wrong. But not everyone is the same, and in healthcare, you know, we don’t really like to think outside the box. It's just part of the health teams, especially when you're just starting out. We don’t like to think outside the box. Like, if there is no bread or milk for breakfast, it’s unhealthy. I think they [the students] were left with the idea that what migrants eat isn’t healthy—simply because no one ever explained it to them. WPDM(1)
4. Burnout among health personnel	60	Listlessness, apathy, and fatigue experienced by healthcare personnel due to systemic stressors in healthcare. This leads to egalitarian care that overlooks cultural aspects, as observed by students in clinical settings	I think that more than discrimination it's a feeling of reluctance. It’s that feeling of, ‘Sigh, here we go again—another patient I won’t be able to communicate with, and it’s going to be tough.’ It’s like this buildup of fatigue, as I see it. It’s exhausting—you have to gesture more, maybe raise your voice, put in more effort than usual—and that wears you down, in my opinion.” WSMi2(2)

**Table 4:** Categories and subcategories related to the materialization of the International Migrant Health Policy in the University Institution Setting

Category 2: University Institution Setting			
Subcategories	Citation Operational	Operational description	Verbatim quotation
Facilitators for materialization			
1. Strategies for integrating content	55	Institutional or program-level strategies that have been put in place to incorporate the PSMI into health sciences curricula	(...)Even though it’s not content that’s included in the curriculum, we're trying to incorporate it (...). For example, in the Family Health course, students are required to analyze and work with a whole family, so it is also a little bit about trying to include families from other countries. Also, some students have done their theses with people from Haiti or other places outside of Chile—basically, people who aren’t Chilean. WPDNu(1)
2. Curriculum renewal	40	Opportunities noticed by academic faculty to incorporate the PSMI into health sciences curricula	Look, since we’re currently in the process of revising the curricula, we’re reviewing each program and making recommendations. So, it’s feasible—perhaps—that we’ll explicitly include this topic, which hadn’t really been considered before, at least not since I’ve been here. But now that we’re working on the redesign, and from the program's point of view, I think it could be a strength and an opportunity to include other topics. MAFNu1(3)
Hindrances for materialization			
1. Minimization of PSMI-related training by PDs	78	Limited value attributed by some PDs to incorporating the PSMI into health sciences curricula because they consider that promoting egalitarian care is sufficient	The patient is treated as a patient, not necessarily as a migrant per se. In other words, regardless of skin color, race, or ideology, a patient is a patient. There shouldn’t be discrimination or special considerations. In other words, they should be treated the same as any other patient. In that sense, that's kind of what we reinforce, especially in the clinical setting—that patients, no matter how they act or look, are still patients. MPDQF(2)
2. Curriculum aspects	69	Different reasons cited by academic faculty as to why PSMI has not been integrated into health sciences curricula include rigid curriculum, curriculum overload, and decontextualized curriculum	(...) I believe these are emerging things [new contents], so there are other things that need to be phased out of the curriculum or covered in less depth because they’re no longer necessary or commonly seen. I went to a CESFAM [Family Health Center] to supervise some students in their rotation, and that's when I realized. I looked at the maternity ward and said, ‘Hey, it’s all foreign people,’ and the student replied, ‘Only she, and she are Chilean.’ The waiting room was full—only two Chilean women among everyone there.” WAFNu1(2)
3. Discrepancy between Institutional Graduate Profile (IGP) and Program-specific Graduate Profile (PGP)	45	Lack of alignment between the IGP and the PGP, where one affirms that it responds to the cultural needs of the population, while the other makes no reference to this aspect	In other words, these competencies have been declared by the university for a long time, respect for diversity and multiculturalism, they have been present for a long time (...) because I believe they were conceived precisely in response to the multiculturalism already present in our country, not specifically because of migrants. However, the academic programs need to respond to these competencies since they’re meant to be achieved by students as part of their graduation profile. What may be missing is making them more explicit in course syllabi and in the program’s overall profile. WPDN(2)
4. Academic bubble	35	Academic isolation that produces a disconnection from contextual realities so that no importance is given to the PSMI	“I haven’t seen any Haitians here, there just aren’t any. Honestly, I haven’t seen them anywhere around, so we don’t have that language problem. Maybe in Santiago there are more, but not here. So, at this university—and specifically in this program—we could start thinking about it for the future, but this isn’t really a city where many Haitians arrive. (...) So, it’s not yet a problem that needs solving or something we need to address right now. I’m not sure if I’m being clear, but it’s just not a priority at the moment.” MPDK (3)

PSMI: International Migrant Health Policy

**Table 5:** Categories and subcategories related to the materialization of the International Migrant Health Policy in the Degree Program Setting

Category 3: Degree Program Setting			
Subcategories	Citation Operational	Description	Verbatim quotation
Facilitators for materialization			
1. Student self- management	83	Autonomous learning strategies that the students resort to when they become aware of the high migrant population they find during clinical rotations, in order to respond appropriately to the needs of the context	It's a reality they [the students] experience every day since we’ve had them doing clinical rotations in primary care starting in their third year. They see that large number of migrants—migrant women—they have to care for and become aware of their needs. They get really interested in the topic right away. They'll say things like: “'Miss, I want to go on a home visit,' or 'I want to do this' or 'I want to try that' (…). They’re super motivated, really receptive, and very collaborative. When you ask them to find some information, they’re more than willing to do it. The other thing is they pick it up quickly and apply it right away in their interventions when they’re on rotation —they’re quick to address the needs they [migrants] may have. WCPMi1 (3)
2. Implicit incorporation of PSMI in training	78	Curricular content currently found in health sciences curricula that contributes, to some extent, to the PSMI	But this kind of development can be reflected in students' achievement of certain competencies. In fact, in the course I teach, —Bioethics and Legal Aspects—as well as in Public Health, which are subjects that are at the same level in the study plan, we really emphasize respect for human beings, their dignity, and their human rights. Maybe we do not address the policy itself —the migrant health policy— but we do talk about sexual and reproductive health, sexual and reproductive rights, and how those must be respected. We approach it from ethical principles —like justice—that every person, no matter their condition or characteristics, deserves respect simply because they’re persons." MPDMi(3)
Hindrances for materialization			
1. Ethnocentric professional training	196	Professional training in health sciences that promotes egalitarian healthcare established by the dominant group	Structurally, in the theoretical part—especially during undergrad—I don’t remember anyone ever telling us, ‘This is how you should treat immigrants.’ And that’s the issue—it’s a real part of our reality, it always has been, but it’s just never been acknowledged. That’s the truth. WSD8 (2)
2. Theory-practice disruption in interculturality (IC)	152	Professional training that delivers theoretical content on interculturality, without corresponding practical application of the theory, which generates a perception of incapacity in the students	Precisely, most of the time, it’s them [the students] who point out that this element—interculturality— this concept isn’t really being applied or put into practice. We go over the theory with them, and that’s when you start thinking, ‘Well, maybe we need to create more practical, applied opportunities.’ Not just through national-level initiatives, but also by integrating aspects of the populations that are becoming part of our country—so that they’re actually included in healthcare. For example, with the Venezuelan and Haitian communities, which we’re seeing more and more in healthcare settings, they [the students] are starting to ask themselves the same questions. WAFN2(2)
3. Lack of AF or CPs trained in PSMI	125	Lack of AF or CPs trained in PSMI, limiting their ability to carry out effective didactic transposition for students	(...) I think it’s important to train our colleagues and keep updating, right? Because I believe there’s still a stigma—it feels like something that’s pretty widespread. Even though there’s been some training and progress, I think there’s still things missing. What about patients who don’t speak Spanish well? There are no translators in any of the CESFAMs [family health centers]. How are we supposed to care for them? How can we provide quality care? The other day, I had a patient from Japan, and luckily, she understood some Spanish. But the appointment that was supposed to take one hour ended up taking two— even using a translator! Thank God there are phones. WCPMi1(3)
4. Minimization of PSMI-related training by AF or CPs	84	Limited value attributed by some AF and CPs to incorporating the PSMI into health sciences curricula because they consider that promoting egalitarian care is sufficient	I think maybe our wrongdoing is that we don't have it in the curriculum. Maybe we just assume it’s already covered, implicitly. For example, when we come across someone from another country, we try to give students other tools—like being humane, being empathetic, and maintaining effective communication—and that applies regardless of the patient’s nationality. I don’t know, to me, those values are what matter most WAFK2(3)
5. Discretionary incorporation of PSMI content by AF	77	Methodological strategies employed by certain AF—typically those in Public Health—to incorporate PSMI content into the curriculum	(...) when I came back [from maternity leave], I realized the increase in foreign population that our country was facing. So, it became clear to me that I had to incorporate this aspect into my courses somehow—because it’s something that’s going to lead to change or evolution in health professionals. It shifts the social dynamic of our users—they’re not the same as before. They come under other circumstances. So, we also have to learn to adapt and accept it. And considering how closed-off our population can be as a country; many times, people see foreigners as invaders. WAFK2(1)

PSMI: International Migrant Health Policy

In line with the methodological approach and based on the preceding descriptive analysis, we proceeded with a hermeneutic analysis. With this analysis it was possible to relate the different facilitators and hindrances identified across the three settings in which students engage during their professional training. 

**Figure 1:**
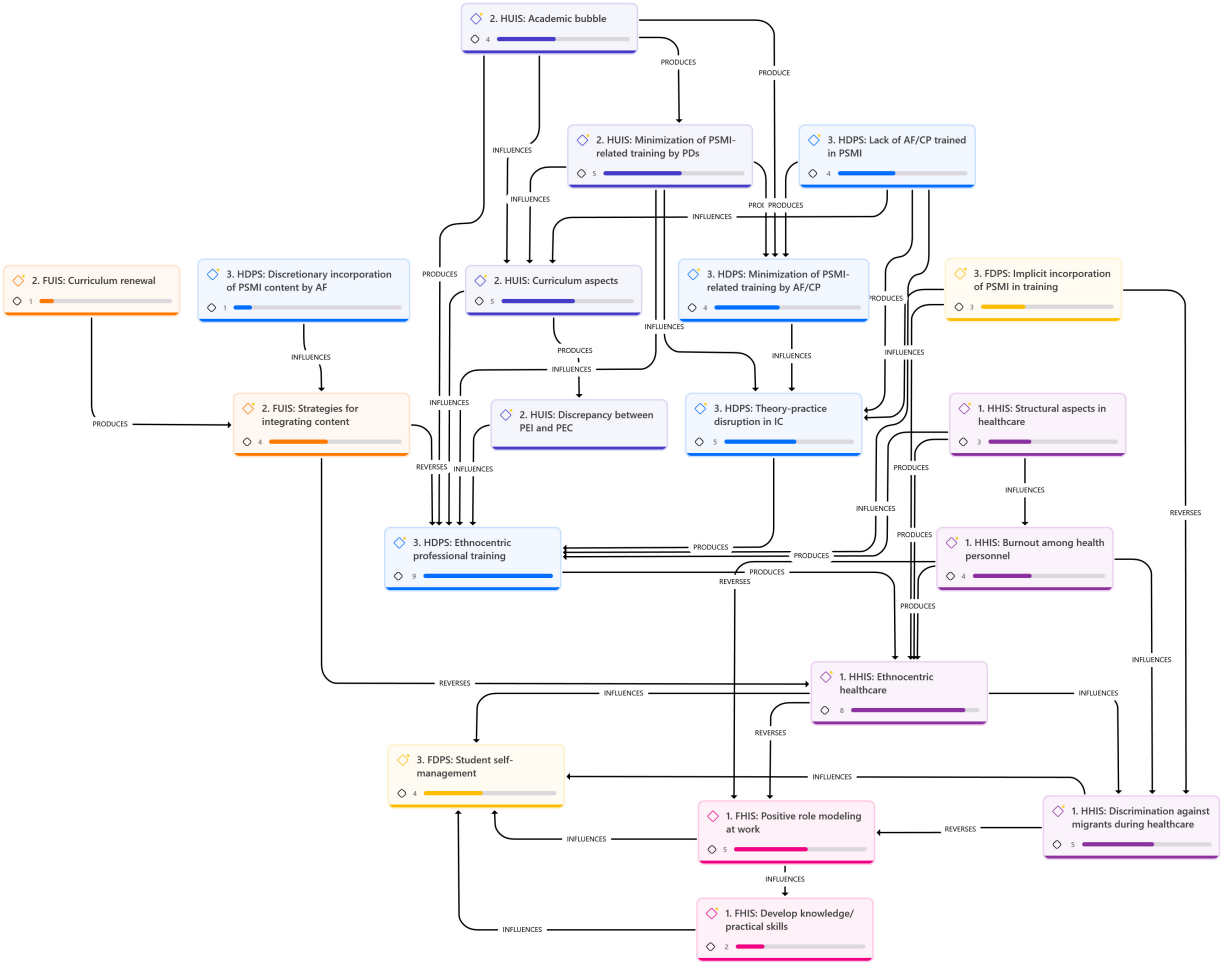
Interrelationship of facilitators and hindrances across Health Institution, University Institution, and Degree Program Settings

*Note: FHIS = Facilitators in the Health Institution Setting; HHIS = Hindrances in the Health Institution Setting; FUIS = Facilitators in the University Institution Setting; HUIS = Hindrances in the University Institution Setting; FDPS = Facilitators in the Degree Program Setting; HDPS = Hindrances in the Degree Program Setting.*

Figure 1 shows the interrelationships and how they may influence, facilitate, or reverse the materialization of the PSMI in health sciences curricula. Overall, facilitators appear scarce, limited, and isolated from the core of the semantic network, with few nodes. In contrast, hindrances are more numerous, with denser and more robust semantic networks and nodes. This results in ethnocentric professional training, which is further reinforced by ethnocentric healthcare practices in clinical settings. Thus, during professional training, homogeneous and universal knowledge is learned without delving into the implications of cultural variability in healthcare. This learning is reinforced and validated during clinical rotations, where students observe ethnocentric healthcare characterized by egalitarianism and standardization.

## Discussion

This research shows that the professional training in health sciences programs in Chile does not adequately respond to the PSMI. The hindrances identified across the Health Institution, University Institution, and Degree Program Settings contribute to the ethnocentric model of professional training, which is supported by the ethnocentric healthcare practices that prevail in clinical settings. While certain facilitators show potential to counteract this trend, they are not powerful enough to reverse the ethnocentric pattern. 

The specialized literature in the health field refers to this phenomenon as cultural blindness, where health personnel are unable to recognize the patient's cultural needs during the care process and, as a result, fail to incorporate alternative perspectives^39^. However, this study highlights that while participants acknowledge the need and importance of integrating patients' cultural backgrounds into healthcare, not all are able to do so due to their numerous hindrances. This finding leads us to propose that it is not "cultural blindness," but rather "cultural blinders." This term was found in social sciences research^40^, but no further studies have been found that developed or established this construct. 

Blinders, also known as blinkers (anteojeras in Spanish), are leather shields placed beside horses’ eyes to keep them focused straight ahead and prevent them from looking sideways so they are not distracted. Drawing an analogy from this equestrian accessory, we can argue that ethnocentric professional training and ethnocentric healthcare contribute to cultural elements being ignored or rendered invisible in the care of migrant patients. However, there is an awareness that this should not happen. Thus, "cultural blinders" could be defined as providing equal and ethnocentric healthcare to people from other cultures despite being aware of the cultural elements involved; however, these elements are not integrated due to structural and personal barriers. 

This way of proceeding may be related to the fact that, currently, training in the PSMI within health sciences programs is largely implicit and often addressed through bioethical and legal content with a human rights approach, which recognizes the equality of individuals before the law and the idea that everyone should be protected from discrimination. However, this perspective reinforces a universalist education that perpetuates an ethnocentric focus, making cultural variability invisible. In this sense, it promotes a view of the other as someone equal to the self, sharing the same characteristics, behaviors, and ways of reasoning^41^. Therefore, for participants, providing equal healthcare is not perceived as wrong. 

Professional training that views the other as equal to the self reflects a form of technical and practical reflexivity within health sciences curricula, which is consistent with other international studies^42^,^43^. There, the same pattern of care is applied to all individuals, regardless of their culture, to comply with protocols, regulations, and standardizations established by the Ministry of Health. As a result, incorporating cultural elements is neither indispensable nor relevant. However, providing truly intercultural healthcare requires developing a critical perspective, as well as including alternative notions of the other in professional training—such as those proposed by Lévinas. 

This author recognizes the other as different from the self, possessing valid particularities that must be acknowledged to provide what the other desires and needs^44^. His premises, based on concepts such as the self, the other, the face, ethics, and the infinite, seek to understand the other through their perspective and within their context^45^. Positioning Lévinas' ethics of otherness into health sciences teaching would allow understanding and taking responsibility toward the other from a critical, rather than moralistic, approach. Achieving this would require shedding the latent sense of Western superiority and totalizing thought to learn to value difference and affirm the dignity of the other. 

Giroux and McLaren argue that individuals should become aware of how dominant groups have constructed and legitimized knowledge over time to serve personal interests. This awareness requires an emancipatory thinking that seeks truth to achieve social justice^46^. In a similar vein, Wenniserí:iostha et al.^47^ advocate for reflection on professional practice as a way to dismantle entrenched boundaries and promote decolonization in healthcare through innovative approaches that reverse the current status quo. 

The results of this research show a lack of critical perspective across the entire health structure —comprising the Health Institution, University Institution, and Degree Program Settings. This absence may underlie the persistent inertia in healthcare, conceptualized here as "cultural blinders," which shape egalitarian, universalist, and ethnocentric care. This is directly related to the minimization stage described by Bennet^48^, in which the participants recognize the existence of cultural differences in care provision for migrant patients but choose to remain neutral, delivering uniform care to maintain equality. As a result, there is a strong likelihood that this professional behavior will be reproduced across future generations by learning through modeling^26^,^28^. 

National empirical evidence shows that training in ICC has been developed for healthcare professionals; however, it focuses on migration-related policies and regulations, migrant rights, and raising awareness about discrimination^49^. No training programs were found that address the development of awareness of the other, a step that would allow the development of cultural openness and lead to cultural inclusion. Nor were there training programs to gain praxeological knowledge that critically examines care practices themselves, as other authors point out^31^,^46^,^47^. 

Countries with greater experience developing intercultural competence (ICC) have implemented concrete measures with short-, medium-, and long-term goals to ensure healthcare is delivered from an intercultural perspective^21^,^50^,^51^. These efforts begin with national policies that explicitly state that, for medical practice, professionals must possess the competencies and skills necessary to respond to population diversity^50^. As a result, higher education institutions have been compelled to design spiral curricula that incorporate content related to historically overlooked topics—such as disability, gender, ethnicity, generational and socioeconomic differences, migration and refugees, and other minority groups—depending on the specific context^50^,^51^. 

The latest research indicates that training in ICC should begin with the development of self-awareness to eliminate existing biases, stereotypes, and prejudices in healthcare toward migrant populations^28^,^29^,^52^. This shift from an ethnocentric to an ethno-relative perspective enables understanding and respecting the other. Other studies are also noteworthy, as they demonstrated the interest and self-management of the new generation of health sciences students to learn about diversity, with the aim of providing inclusive and culturally relevant care^26^,^31^,^53^. This interest should be considered a tremendous opportunity to incorporate ICC into training programs, as it builds on the students' intrinsic motivation. 

As a research team that has been studying this topic for over five years, we recognize that designing a curriculum in intercultural competence (ICC) requires more than the interest and willingness of different health professions. It also requires considering insights from disciplines such as philosophy, law, medical and social anthropology, sociology, linguistics, and psychology to examine the implications of these conceptual frameworks in healthcare processes. Incorporating only clinical aspects can contribute to the development of stereotypes and biases. 

Consequently, for health sciences curricula to respond to the PSMI, it is urgent to create joint public policies between the Ministry of Education and the Ministry of Health. The Ministry of Education should establish specific accreditation criteria for health-related degree programs to ensure professional training in ICC to provide culturally relevant care. Meanwhile, the Ministry of Health should create professional profiles that require professional ICC training as a prerequisite for entry into the workforce. Likewise, quality of care criteria must be created to ensure cultural safety. This will also involve the creation of Intercultural Mediation Units in healthcare centers across the country, with an interdisciplinary team that will support clinical healthcare and help resolve ethical dilemmas that may arise in clinical practice. These measures could reverse the prevailing cultural blinders in the healthcare structure that perpetuate ethnocentric care and training. 

The greatest strength of this research is its inclusion of all individuals involved in the teaching-learning process within health sciences curricula, providing an overall view of the phenomenon under study. However, a limitation is that the study only included three universities in Chile. 

Future lines of research should conduct quantitative pre- and post-test or mixed-method studies to evaluate the progress of educational interventions on ICC in health sciences curricula in Chile. In this way, ICC incorporation and development in professional training could be analyzed, and improvements could be proposed during its execution. 

## Conclusions

This study demonstrates that the professional training of health sciences students in Chile does not adequately respond to the materialization of the PSMI. Although facilitators help the policy’s materialization, they are limited in number and confined to enclosed spaces. In contrast, hindrances encompass broader aspects and are more pervasive within the entire healthcare structure, thereby restricting the effectiveness of professional training in ICC. All this makes it possible to propose the cultural blinder construct because the participants are aware that cultural elements are not incorporated or minimized in both professional training and healthcare for migrant patients. Nevertheless, inertia and adherence to the status quo persist, driven by an ethnocentric perspective that equates the other with the self, ultimately rendering cultural diversity invisible.

Therefore, it is essential to explicitly integrate the PSMI into health sciences curricula with a critical and reflective approach, so that future professionals can propose and validate decolonizing healthcare that promotes equity and social justice in a diverse society. 

However, this necessarily requires the development of public policies that link, coordinate and structure the actions of both the Ministry of Health and the Ministry of Education to advocate harmoniously for healthcare that responds to the PSMI. In addition, there is a need to establish precise accreditation criteria to measure the development of ICC within health sciences curricula.
